# MicroRNA-27a (miR-27a) in Solid Tumors: A Review Based on Mechanisms and Clinical Observations

**DOI:** 10.3389/fonc.2019.00893

**Published:** 2019-09-12

**Authors:** Jingcheng Zhang, Zhe Cao, Gang Yang, Lei You, Taiping Zhang, Yupei Zhao

**Affiliations:** ^1^Department of General Surgery, Peking Union Medical College Hospital, Chinese Academy of Medical Sciences and Peking Union Medical College, Beijing, China; ^2^Peking Union Medical College, Chinese Academy of Medical Sciences and Peking Union Medical College, Beijing, China; ^3^Clinical Immunology Center, Chinese Academy of Medical Sciences and Peking Union Medical College, Beijing, China

**Keywords:** microRNA, microRNA-27a, signaling pathways, biomarker, clinical application

## Abstract

MicroRNAs (miRNAs) are a family of highly conserved, non-coding single-stranded RNAs transcribed as ~70 nucleotide precursors to an 18–22 nucleotide product (1). miRNAs can silence their homologous target genes at the post-transcriptional level, and these genes have been revealed to play an important role in tumorigenesis, invasion and metastasis (2). MicroRNA-27a (miR-27a), transcripted by miR-27a gene, has proved to implicate with many kinds of solid tumors, showing potential as a useful biomarker or drug target for clinical application. However, even though miR-27a has been reported in many cancers, the mechanism and signal pathways of miR-27 in oncogenesis, invasion, and metastasis are still obscure. Moreover, recent studies show that miR-27a pays an important role in epithelial-mesenchymal-transition, regulating tumor immune response, and chemoresistance. In this review, we summarize the current literature, demonstrate the established link between miR-27a and tumorigenesis, and focus on recently identified mechanisms. The review also aims to demonstrate the potential of miR-27a as a diagnostic and/or prognostic biomarker in solid tumors and to discuss the possibilities of targeted therapy and drug design.

## Introduction

Since microRNAs (miRNAs) were discovered in *Caenorhabditis elegans* as important non-coding (18–22 nucleotides, nt) RNA molecules, they have attracted the attention of many researchers for their multifaceted roles in controlling cancer development by regulating target genes (3–5). miRNAs show variable distribution and expression features in different kinds of solid cancer cells, showing diverse regulatory mechanisms for deep research (6). After decades of progress, hundreds of miRNAs, beginning with line-4 miRNA to let-7 miRNA to the miRNA family, were discovered, and their functions in tumor cells have been partially explained. MicroRNA-27a, transcribed by the miR-27a gene on chromosome 19p13.13, is an important member of the miRNA family. The mature miR-27a is 22 nt long and forms one strand of the RNA duplex. With the cooperation of the RNA-induced silencing complex (RISC), miR-27a can exert different regulatory functions in different kinds of cancer. miR-27a was first implicated in breast cancer, in which high miR-27a expression increased the percentage of cells in G2/M stage, resulting in an oncogenic function (7). miR-27a was also found to be upregulated in ovarian cancer cells and prostate cancer cells and showed good potential for therapeutic applications (8, 9). In recent years, because of the heterogeneity and variability of genetic expression features of many specific oncogenes or tumor suppressors in gastrointestinal cancer, miR-27a has also been studied in gastrointestinal cancer, opening a broad area for research. For example, miR-27a is upregulated in gastric adenocarcinoma, while the suppression of miR-27a can reestablish the sensitivity of tumor cells to chemotherapeutic drugs (10, 11). It has been concluded that miR-27a plays an important role in oncogenesis, cell proliferation, tumor cell metabolism, and chemotherapy resistance. In recent years, with the further recognition of tumor biogenesis, miR-27a shows novel functions in regulating the tumor immune response and epithelial-mesenchymal transition (EMT) (12, 13). With further research on miRNA exosomes, miR-27a showed good potential use in the clinical therapeutic or prognostic areas (14). However, although miR-27a has been shown to have important research value, we found that studies on miR-27a in various tumors are diverse, and studies on the mechanisms and signaling pathways of miR-27a are inadequate. Clinical studies on the use of miR-27a as a diagnostic or prognostic biomarker are also needed. In this review, we summarize the recent developments in miR-27a research, analyze the biogenesis and functional features of miR-27a, focus on the key signaling pathways affected by miR-27a in cancer cells, summarize recent clinical research on miR-27a as a biomarker or therapeutic target, and provide directions for further studies.

## Biogenesis of miR-27a in Solid Tumors

**Figure 1:**
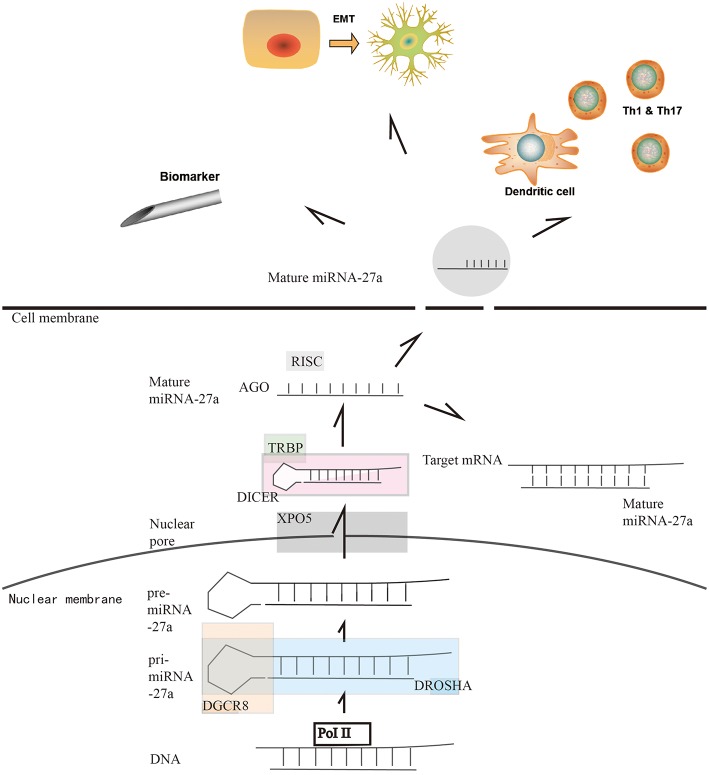
Overview of microRNA-27a biogenesis, highlighting key enzymes in miRNA biogenesis. miRNAs are produced in a tightly regulated pathway which is conserved across species. As a canonical biogenesis progress, the biogenesis of miR-27a begins with their transcription by RNA polymerase II (Pol II). Following RNA Pol II-mediated transcription, the first of two enzymatic cleavages produce pre-miRNAs. Drosha, a type III RNase, along with the cofactor DGCR8, binds to the primary miRNA (pri-miRNA) transcript. Two RNase domains that are present in Drosha mediate the cleavage of the 3′ and 5′ strands of pri-miRNAs to generate pre-miRNA (16). Next, the exportin 5–RAN∙GTP complex (XPO 5) mediates the movement of pre-miRNAs from the nucleus into the cytosol. There, the RNase III Dicer combinding with TAR RNA binding protein (TRBP) binds to the pre-miRNAs and cleaves the terminal loop, producing a miRNA duplex. Then, incorporated into the RNA-induced silencing complex (RISC), miRNA duplex is processed by the argonaute (AGO) family of proteins to get the matured single-strand miRNA. For the function of miR-27a, on the one hand, the main function of the miRNA is that binding to selected mRNA to form a slienced mRNA duplex. On the other hand, miR-27a can form exosomes to release in to bloodstream. It is reported that miR-27a can regulate the immune system by affecting dentritic cells and macrophages. It is also reported that the exosomal miR-27a can regulate the EMT of cancer cells. Furthermore, the exosomal miR-27a in the bloodstream gives us new method of early diagnosis of cancers.

The canonical biogenesis of a miRNA has been defined and explained by previous research (3, 15). In cancer cells, the well-regulated miRNA biogenesis pathways are dysregulated since there is a shift in gene expression and dysregulation of the key enzymes, leading to the up/downregulation of different miRNAs in cancer cells. These pathways and key enzymes, such as Drosha, exportin 5, DICER and Argonaute 2 (AGO2), are briefly described in Figure 1 (3, 15, 17–19). Studies on miR-27 have indicated that the miR-27 family is a large family and that miR-27a is one of the products of the gene loci. There are several isomiRNAs of miR-27 formed in the mature progress derived from alternative and imprecise Drosha and DICER cleavage that have different expression levels and functions. In liver cancer, miR-27a-3p has a high expression level of isomiR in normal cells, while isomiR expression is decreased significantly in tumor cells. However, this differentiation is not observed at the homologous miR-27b-3p site, indicating that isomiR-27a may have a more complicated background in tumor cells (20). As a 22-nt long mimic, it is easy for miR-27a to cooperate with other miRs. It has been revealed that miR-27 operates together with miR-23 and miR-24 in a cooperative cluster since these miRNA gene clusters are located on chromosome 19(–) (21). Therefore, some studies have focused on the function and clinical use of the miR-23a-27a-24-2 cluster instead of miR-27a, which requires further discussion. In addition, it has been proven that herpesvirus saimiri expresses several non-coding RNAs that significantly reduce the level of miR-27 in a host cell, indicating an interruption of miRNA biogenesis (22). In gastrointestinal tumors, with the variable expression level of different key enzymes, miR-27a has been discussed in specific kinds of tumors, while miR-27a has shown different expression features in gastrointestinal tumors. In esophageal tumors, DROSHA/DGCR8 is upregulated, corresponding with the upregulation of miR-27a in tumor cells (23). In gastric cancer, DROSHA and DICER are upregulated, indicating a high level of miR-27a in the cytoplasm (24). It was also reported that DGCR8 and DICER were upregulated in colorectal carcinoma (25, 26). These studies indicate that miR-27a is highly expressed in gastrointestinal tumors. However, in hepatic cancer cells, a researcher found the downregulation of DICER, which indicates a lower level of miR-27a (27). The molecular and cellular pathways controlling miRNA biogenesis remain to be further studied. In 2019, one novel study showed that melanoma differentiation-associated gene-7/interleukin-24 (mda-7/IL-24), a multifunctional cytokine, displayed broad-spectrum anticancer activity by regulating the function of DICER. This study provides a new anticancer method in which key miRNA enzymes, such as DICER, are targeted to disrupt miRNA synthesis in cancer cells (28).

In recent years, the distribution of miRNAs in the serum or intracellular space, including exosomes, has attracted great attention from researchers as an important feature of miRNA biogenesis because miRNAs can be used as predictive or prognostic biomarkers. Regarding miR-27a, there have been several studies on the relationship between the serum level of miR-27a and patient prognosis. miR-27a has been proven to be a predictive or prognostic biomarker in gastrointestinal tumors in recent years since miR-27a can be secreted by exosomes from cells into serum circulation in gastric cancer. It has also been reported that circulating exosomal miR-27a can be used as a novel diagnostic and prognostic biomarker in colorectal cancer and hepatitis C virus-associated hepatocellular carcinoma (HCC) (29–33). In pancreatic cancer, although there was no specific finding on exosomal miR-27a, there was an increase in miR-23 exosomes, which can be used as a biomarker (14, 34, 35). Considering the miR-23-27-242 cluster, the miR-27a exosome might be a possible research direction in pancreatic adenocarcinoma. Further functions of exosomal miR-27a, such as the regulation of cancer-associated fibroblasts, will be reviewed in the following chapter.

## Roles of miR-27a in Regulating Signaling Pathways

### miR-27a in Regulating AKT Signaling Pathways

microRNA-27a is involved in mechanisms related to proliferation and growth signaling pathways (18). miR-27a promotes cancer cell proliferation by repressing the expression of prohibitin, which can block E2F to interrupt the AKT or tyrosine signaling pathways (10, 11, 36). Furthermore, it has been reported that the pleckstrin homology (PH) domain and leucine-rich repeat protein phosphatase 2 (PHLPP2) are new targets of miR-27a. The miR-27a could induce suppression of PHLPP2, leading to stimulation of the AKT/GSK3β pathway (37). FOXO1 is an important transcription factor downregulated by AKT directly, while adipose tissue-secreted miR-27a promotes liver cancer by downregulating FOXO1 (38). PI3K is a novel target of miR-27a. By activating the PI3K/AKT signaling pathway, the phosphorylated survival protein AKT is strongly expressed. The upregulation of miR-27a targets PI3K, initiating apoptosis (39). Bcl-2, a key target in the PI3K/AKT and JNK pathways, is a direct target of miR-27a, and overexpression of Bcl-2 attenuates the promotion of cell damage by miR-27a (40). In addition, down-regulation of miR-27a significantly reduced the expression of cyclin D1 transcriptional activity and up-regulated the expression of p21 downstream of GSK3β (11). This evidence indicates that miR-27a regulates proliferation and growth by interacting with the AKT pathway.

### miR-27a Regulates the Wnt/β-catenin Signaling Pathway

Recent studies have shown that miR-27a can directly target secreted frizzled-related protein (SFRP), a regulator of tumor suppressor proteins and Wnt signaling pathway. miR-27a is overexpressed in cancer cell lines (MGC803, BT-20, MCF-7, T-47D, and MDA-MB-231). When miR-27a is downregulated, SFRP1 is upregulated, and β-catenin, Wnt, pβ-catenin, and p-Wnt are significantly downregulated at the same time. In contrast, when transfected with miR-27a mimics, the proliferation, migration, and invasion of gastric cancer cells are remarkably increased, accompanied by a decrease in SFRP1 protein, indicating the inhibition of SFRPs and continuous activation of the Wnt/β-catenin signaling pathway (16, 41). Up-regulation of obesity-related miR-27a can also promote HCC metastasis by inhibiting SFRP1 (42). In another study, the authors found that miR-27a negatively regulates the expression of SFRP1 mRNA. HCT-116 cells transfected with miR-27a mimics show increased proliferation and invasion of colon cancer cells, while the expression of pβ-catenin is remarkably downregulated (43). In addition to the SFRP family, RARalpha-mediated transcriptional inactivation of miR-27a activates the inhibition of GSR-3β by miR-27a, which leads to cancer differentiation via the Wnt/β-catenin pathway involved in GSK-3β (44). RXRalpha is a target gene of miR-27a-3p (45). Transmembrane protein 170B (TMEM170B) is also revealed as a novel functional target of miR-27a, which promotes cytoplasmic β-catenin phosphorylation, leading to inhibition of β-catenin stabilization (46). miR-27a upregulation and the activation of PPARgamma/β-catenin signaling were verified by recent research (47). Moreover, miR-27a promotes EMT through Wnt/β-catenin signaling (12, 48). Thus, the Wnt/β-catenin signaling pathway is an important target pathway for miR-27a.

### miR-27a in Regulating the Ras/MEK/ERK Signaling Pathway

Another study found that two isoforms of mature miR-27a, miR-27a-5p, and miR-27-3p, have different expression levels in gastric cancer: miR-27-3p expression is significantly higher than that of miR-27a-5p. By inhibiting B cell translocation gene 2 (BTG2), miR-27-3p can upregulate the Ras/MEK/ERK pathway and c-myc levels, promoting cancer cell proliferation (49). Novel research has shown that miR-27a promotes ERK phosphorylation by downregulation of the ERK inhibitor sprouty2 (50). It has also been proven that miR-27a regulates the p38/mitogen-activated protein kinase (MAPK) signaling pathway (51). Furthermore, miR-27a directly silences the FBXW7 gene by binding to its 3′ untranslated region (UTR), thereby reducing the expression of FBXW7 in cancer cell. Silencing of FBXW7 in turn further increases the expression of KLF5 and miR-27a, leading to the dysfunction of c-myc (52, 53). Under the regulation of c-myc gene, the expression of mature miR-23a, miR-24-2 and miR-27a is promoted, thereby subsequently decreasing the expression of SPRY2 and activating p44/42 MAPK to promote cancer cell invasion (54). Taken together, these results suggest that miR-27a also has an effect on the MAPK or RAS/MEK/ERK pathway by regulating several genes.

### miR-27a in Regulating the TGF-β Signaling Pathway

miR-27a promotes tumor proliferation and invasion by inhibiting TGF-β-induced cell cycle arrest. Overexpression of miR-27a can reduce SMAD2 and SMAD4 at mRNA and protein levels, which has important tumor suppressive effects in TGF-β signaling pathway (55, 56). miR-27a can also directly target the 3′-UTR of transforming growth factor β receptor I (TGF-βRI) and downregulate TGF-β signaling (57). By repressing PPARgamma, miR-27a activates TGF-β/Smad3 signaling and contributes to the changes in the expression of connective tissue growth factor (CTGF) (58). miR-27a acts as an oncogene by silencing MAP2K4, which is an important tumor suppressor inhibiting cell proliferation and migration via the JNK/p38 signaling pathway downstream of the TGF-β/TAK1 pathway (59).

**Figure 2:**
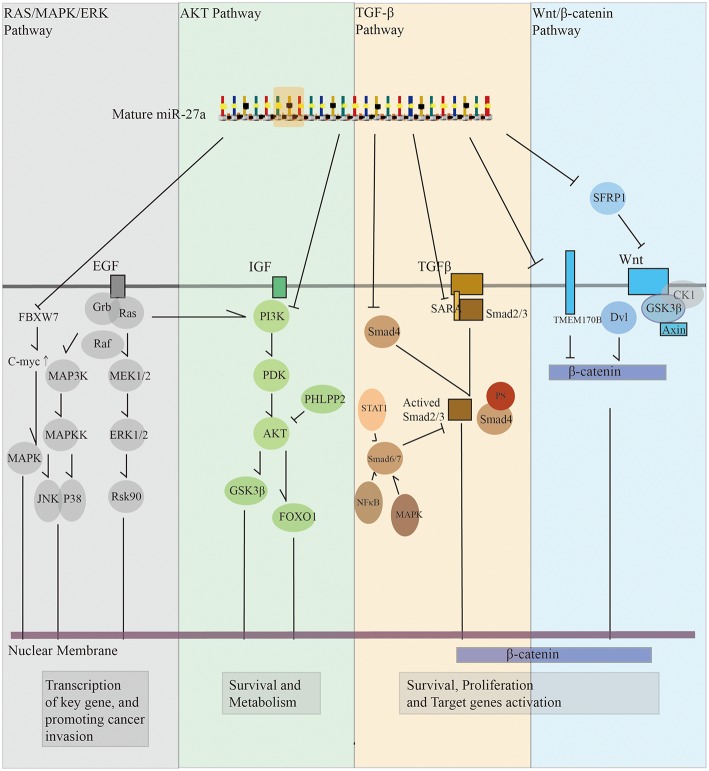
A brief conclusion of miR-27a in regulating cell signal pathways Panel gray, miR-27a can regulate Ras/MAPK/ERK pathway to active key gene such as c-myc. JNK and P38, which pay important role as a downstream of TGF-13 signal pathway, are also involved in Ras/MAPK cascade pathway. c-myc is also plays an important role in this progress. Panel green, miR-27a can regulate AKT pathway to keep the activation of survival signal of tumor cells. Several key enzymes in AKT pathway such as PI3K, GSK3J3, and FOXO1 are reported as a direct target of miR-27a. Panel yellow, miR-27a can decrease SMAD2 and SMAD4 mRNA and protein levels to regulate TGF-I3 signal pathway. Panel blue, by depressing TMEM170B and SFRP1, miR-27a leads to aberrant activation of Wnt/J3-catenin pathway to promote the proliferation and survival of cancer cells.

We summarize four important signaling pathways that are affected by miR-27a directly or indirectly (Figure 2). Although there is overlap between the signaling pathways, we can conclude that miR-27a has extensive effects on the mainstream signaling pathways, which brings us more confidence in understanding the function of miR-27a in tumorigenesis and converting basic medical research into clinical use.

## miR-27a in Promoting Tumorigenesis

### miR-27a Has Different Functions in the EMT of Different Solid Tumors

EMT is an important aspect of metastasis that enables cells to migrate and populate secondary sites, and microRNAs play an important role in the EMT of tumors (60, 61). In solid tumors, such as breast cancer, gastrointestinal cancer, and ovarian cancer, transformed cells that undergo EMT lose epithelial features and acquire mesenchymal features for invasion and migration (62). EMT is performed by the so-called EMT-activated transcription factor (EMT-TF), mainly the SNAIL1, TWIST, and ZEB families (63). A recent study showed that EMT-TF can be dynamically degraded by the atypical ubiquitin E3 ligase complex Skp1-Pam-Fbxo45 (SPFFbxo45) via the ubiquitin-proteasome system (UPS). Fbxo45 recognizes EMT-TF by Zeb2 through its SPRY domain is a key step in this process, while miR-27a can directly down-regulate the expression of Fbxo45, preventing EMT-TF degradation and ensuring EMT (64). Several key signaling pathways that are highly regulated by miR-27a as mentioned before, such as the Wnt/β-catenin pathway and GSK3β, have been proven to participate in the regulation of EMT (43, 45, 48, 65). In a novel study, the mRNA expression level of miR-27a, which activates the Wnt/β-catenin signaling pathway through the inhibition of FOXO1, was significantly higher in ovarian cancer tissues and in *in vitro* ovarian cancer cell lines (12).

Moreover, another novel study found that AP-2β, an important transcription factor and a tumor suppressor, is inhibited by miR-27a in HCC. AP-2β modulates the levels of EMT markers through Slug and Snail. miR-27a reverses the tumor suppressive role of AP-2β by binding to the AP-2β 3′-UTR (66). Thus, a miR-27a/AP-2β/Slug/EMT regulatory axis may be a potential target for evaluating treatment and prognosis (66). miR-27a also shows heterogenic functions in different types of cells. In HCC, miR-27a-3p downregulation contributes to a higher level of Twist-1 and Bcl-2 expression, causing vasculogenic mimicry and metastasis by downregulating VE-cadherin expression, indicating the inhibition of EMT and early metastasis (67–69). miR-27a-3p may also have an antitumor function in the development of HCC by targeting dual specificity phosphatase 16 (DUSP16) to decrease the viability and migration of tumor cells (70). F-box and WD repeat domain containing 7 (FBXW7), which notably suppresses EMT and migratory activity, is a downstream target gene of miR-27a. miR-27a can inhibit FBXW7, leading to the dysregulation of downstream genes and the activation of EMT (71).

### miR-27a Plays an Important Role in Regulating Cancer Stem Cells (CSCs) and the Tumor Microenvironment (TME)

In recent years, the functions and regulatory mechanisms of CSCs have attracted considerable attention. There is some evidence that CSCs are dynamic in many cancer types, while EMT-TFs, such as TWIST1 and ZEB1, play an important role in regulating CSC properties (72–74). The interconversion of CSCs and non-CSCs was also considered as a relatively common phenomenon (75). Recent studies have shown that, suggesting that the tumor endothelium may be derived from CSC (76). In breast cancer stem-like cells (BCSLCs), up-regulation of miR-27a and promotion of angiogenesis were observed after treatment of BCSLC with vascular endothelial growth factor (VEGF) (77). In a non-small cell lung cancer (NSCLC) stem-like cell line (H1650 CD133(+) CD34(–) cells), researchers have found that miR-27a has higher expression in H1650 CSCs and regulates cancer development in H1650 cells (78). In contrast, another study showed that the downregulation of miR-27a enhances the stem-like properties of small cell lung cancer (SCLC) cells *in vitro* (79).

The TME is described as a combination of the stroma, extracellular matrix elements, and immune cells that have an important role in cancer invasion, migration, and metastasis (80, 81). Carcinoma-associated fibroblasts (CAFs) are a highly enriched cell population in the TME that plays an important role in cancer invasion (82). A novel study showed that exosomal miR-27a in gastric cancer can induce the transformation of fibroblasts into CAFs. Moreover, CAFs overexpressing miR-27a could increase the malignant behavior of gastric cancer cells (29). Several studies have shown that miR-27a is overexpressed in liver stellate cells, influencing fat accumulation and cell proliferation, leading to hepatic fibrosis (83). It was also proven that miR-27a can induce liver fibrosis and be used as a diagnostic biomarker in advanced liver fibrosis and cirrhosis with hepatitis viruses B and C (84).

miR-27a also regulates the immune system to avoid the immune destruction of cancer cells. First, miR-27a can regulate T cell immunity. By accumulating TGF-β, miR-27a is upregulated and then activates the downstream NF-kappaB and MAPK pathways that influence the production of proinflammatory cytokines, leading to the decreased dendritic cell-mediated differentiation of Th1 and Th17 cells, which are important in the tumor immune response (13). Likewise, TGF-β upregulates the expression of the cluster in CD8(+) T cells (85). Second, miR-27a regulates macrophages to promote cancer progression. For example, the miR-23a/27a/24-2 cluster is significantly decreased in the tumor-associated macrophages (TAMs) of breast cancer patients while macrophages overexpressing the miR-23a/27a/24-2 cluster inhibit tumor growth (86). miR-27a also regulates the inflammatory response of macrophages by targeting IL-10 (87). In addition, the miR-23a/27a/24-2 microRNA cluster inhibits B cell development, which indicates a suppression in B cell immunity (88). Taken together, these and other studies suggest that miR-27 plays an important role in regulating CSCs, activating CAFs, and suppressing the immune system to avoid the immune destruction of cancer cells.

### miR-27a and Therapy Resistance

Chemoresistance is frequently observed in most cancers, such as lung cancer and gastric cancer (89, 90). miR-27a has been reported to be associated with chemotherapy resistance in several cancers, while the mechanism of miR-27a in chemoresistance, despite several years of research, remains unclear (91). Multidrug resistance gene-1 (MDR1) is a well-studied gene in chemoresistance. The expression levels of MDR1 mRNA and P-glycoprotein (P-GP) and homeodomain-interacting protein kinase-2 (HIPK2) proteins are upregulated after upregulating the miR-27a level in cancer cells, while the sensitivity to paclitaxel is decreased in cancer cells transfected with miR-27a mimics (8, 92). It was also confirmed that the increase in FZD7 by miR-27a promotes the expression of MDR1/P-GP and β-catenin, leading to chemotherapy resistance (93). In gastric cancer, the downregulation of miR-27a concomitant with higher levels of MDR1, HIF1A, and HIPK2 genes indicates chemoresistance (94). In addition, researchers found increased expression of miR-27a in tumor tissues sampled from lung adenocarcinoma patients treated with cisplatin-based chemotherapy, which correlates with low RKIP expression, leading to resistance to cisplatin and a poor prognosis (95). miR-27a also weakens the effect of cisplatin by targeting RKIP in liver cancer cells (96).

One recent study found that a high level of microRNA-27a induces resistance to TNF-related apoptosis-inducing ligand (TRAIL), while the knockdown of microRNA-27a resensitizes colorectal CSCs to TRAIL-induced cell death (97). miR-27a also induces chemoresistance to tamoxifen in human breast cancer cell lines (98). A novel study found that cells show decreased expression of ERalpha and miR-27a. The overexpression of miR-27a increases the level of ERalpha, increasing the sensitivity of tamoxifen-resistant cancer cells to selective estrogen receptor modulator treatments and resensitizing the cells to tamoxifen (99).

In addition to targeting CDC-27, miR-27a and its downregulation also confer increased radioresistance in triple-negative breast cancer (TNBC) cells (100). As mentioned before, the miR-27a-induced transformation of cancer-associated fibroblasts will decrease drug transportation and efficacy by the TME (101).

## miR-27a in Clinical Applications

### miR-27a as a Biomarker

Since miR-27a is closely related to oncogenesis, many studies have been eager to translate it into clinical applications as a biomarker to help with diagnosis and treatment. miR-27a, specifically exosome miR-27a, has been used as a biomarker for diagnostic and prognostic applications in several solid tumors such as gastric cancer, colorectal cancer, and breast cancer (Table 1).

**Table 1:** Diagnostic or prognostic value of miR-27a in different cancers.

**Cancer type**	**Diagnostic/prognostic**	**Method/comparison**	**Result**	**References**
Gastric cancer	Diagnostic	Five-serum miRNA signature including miR-27a/CEA, CA19-9	Sensitivity 0.879 (95% CI 0.822–0.936)/specificity 0.831 (95% CI 0.767–0.898)	(102)
	Predicting lymph node metastasis	miR-27a in lymph node positive patients/negative patients	*P* = 0.003	(103)
	Prognostic	miR-27a expression level/overall survival	HR 1.304 [95% CI 1.031–1.650], *P* = 0.0270	(104)
	Predicting chemosensitivity	miR-27a/partial response rate	7.7% (high expression patients) vs. 25.9% (low expression patients), *P* = 0.018	(105)
	Diagnostic	Serum miR-27a	75% sensitivity/56% specificity	(30)
Colorectal cancer	Diagnostic/prognostic	Four-serum miRNA signature containing miR-27a	89% sensitivity/81% specificity (AUC = 0.922)	(106)
	Diagnostic	Exosomal miR-27a and miR-130a	AUC = 0.801	(32)
Breast cancer	Diagnostic	Four-serum miRNA signature containing miR-27a in TNBC	Sensitivity 0.75/specificity 0.56 (AUC = 0.74)	(107)
Prostate cancer	Prognostic	Five-serum miRNA signature including miR-27a/rate of recurrent after surgery	AUC = 89.5% (95% CI 79.5–99.5%)	(108)
Hepatic cancer	Diagnostic	Combination of miR-27a and AFP	AUC = 87%	(109)
	Diagnostic	miR-27a expression/differentiating HCC from post-hepatitis C cirrhosis	AUC = 0.897	(33)
Pancreatic cancer	Diagnostic/prognostic	miR-27a alone/combination of miR-27a and serum CA19-9	miR-27a: 82.2% sensitivity/76.7% specificity (AUC = 0.840; 95% CI, 0.787–0.885%); combination: 85.3% sensitivity/81.6% specificity o (AUC = 0.886; 95% CI, 0.837–0.923%)	(35)

#### miR-27a as a Biomarker in the Diagnosis and Prognosis of Gastric Cancer

In gastric cancer, there are several studies showing that miR-27a can be a good candidate as a biomarker for diagnosis and prognosis (30, 103, 105, 110, 111). From 164 gastric cancer patients and 127 normal people, miR-27a was identified to have a markedly higher confidence interval (0.879) than those of carcinoembryonic antigen (CEA) (0.503) and carbohydrate antigen 19-9 (CA19-9) (0.600), indicating that miR-27a can serve as a biomarker for gastric cancer diagnosis (102). In a recent study, the miR-27a expression levels were found to be significantly higher in patients with gastric cancer compared to the validation plasma cohort (30). Moreover, the serum miR-27a level could be a predictive marker for lymph node metastasis in gastric cancer (103). miR-27a can also be a prognostic biomarker in gastric cancer, and the significantly upregulated expression of miR-27a highly correlates with a poor prognosis in gastric cancer patients (112). Exosomal miR-27a can be a predictor of the tumor response to treatment or chemotherapy resistance. In a novel study, the serum level of exosomal miR-27a was detected in 74 gastric cancer patients who received neoadjuvant chemotherapy by qRT-PCR, and the expression of miR-27a in the serum of gastric cancer patients decreased significantly after neoadjuvant chemotherapy. Gastric cancer patients who had a higher level of miR-27a tended to have tumor invasion and migration, indicating poor efficacy and prognosis after neoadjuvant chemotherapy (110). In another study, patients with upregulated miR-27a expression had significantly worse overall survival (OS) than patients with lower miR-27a expression (*P* = 0.024), while a higher miR-27a expression level indicated resistance to fluoropyrimidine-based chemotherapy (105).

#### miR-27a as a Biomarker in the Diagnosis and Prognosis of Colorectal Cancer

In colorectal cancer, recent studies have shown good potential for miR-27a as a biomarker for diagnosis and prognosis. Serum samples from 427 colon cancer patients and 276 healthy donors were included in a recent study. The results showed that miR-27a-3p has a sensitivity of 89% and a specificity of 81% in distinguishing colon cancer patients from healthy donors, indicating circulating microRNAs as a highly sensitive, non-invasive early detection method for colorectal cancer (106). Another study in 2018, which included 369 peripheral blood samples, also showed that exosomal miR-27a in the plasma may act as a non-invasive biomarker for early diagnosis. More importantly, this research provided the first evidence that patients with colorectal cancer with high circulating exosomal miR-27a expression had a poor prognosis, which made up for the previous research gap (32).

#### miR-27a as a Biomarker in Other Solid Cancers

Similar to gastric cancer and colorectal cancer, miR-27 shows wide potential for clinical applications in many solid cancers. miR-27a has been well studied in breast cancer as a biomarker for diagnosis and prognosis for breast cancer or TNBC (107, 113–115). The role of miRNAs in resistance to treatment for breast cancer is also one of the core issues discussed at present (116). miR-27a also shows potential in the diagnosis/prognosis of prostate cancer and the identification of metastasis after radical prostatectomy (108, 117). In HCC, compared to that of a single AFP marker, the combination of miR-125b/miR-27a/AFP had a higher sensitivity and specificity for the diagnosis of early-stage HCC (109). Several studies have suggested the potential use of serum miR-27a as a biomarker for HCC diagnosis; however, further studies are needed (33, 118). In conclusion, miR-27a has broad prospects in clinical applications as a biomarker for early diagnosis, prognosis, tumor stage, and the evaluation of chemotherapy efficacy or metastasis.

### miR-27a as a Potential Target for Drug Design

Although many basic studies have revealed that miR-27a may be a promising drug design target, few drugs that target miR-27 have been developed and examined in clinical trials. In 2011, one study found that ethyl 2-((2,3-bis(nitrooxy)propyl) disulfanyl)benzoate, known as GT-094, a nitric oxide-releasing non-steroidal anti-inflammatory drug (NO-NSAID), decreased Sp1, Sp3, and Sp4 expression in colon cancer cells by downregulating miR-27a and inducing ZBTB10, revealing an anticancer function of this compound (119). Moreover, by downregulating miR-27a expression, curcumin and boswellic acid can induce chemoprevention in colon cancer, which provides new ideas for cancer prevention (120). Arsenic trioxide, a widely used anticancer drug in leukemia, was found to suppress cell growth and migration via the inhibition of miR-27a in breast cancer cells, providing a novel antitumor mechanism in miR-27a for the treatment of breast cancer (121). In addition, one novel study showed that liraglutide inhibited the proliferation and promoted the apoptosis of MCF-7 breast cancer cell lines through the downregulation of microRNA-27a expression (122). These studies provide a new method of drug design and a novel use of traditional drugs.

## Discussion

With an in-depth understanding of cancer, several studies on the mechanism of tumorigenesis have been revealed. miRNAs, as important components that regulate the progression, invasion, and metastasis of cancer cells, should not be disregarded for their remarkable applications in the diagnosis and prognosis of cancer. We conclude that miR-27a plays an important role in oncogenesis, cell proliferation, tumor cell metabolism, and chemotherapy resistance. miR-27a also shows novel functions in regulating the tumor immune response, CAFs, and EMT. Previous studies on these basic medical mechanisms of miR-27 have been reviewed (123, 124). In this review, especially, we analyzed the recent application of miR-27a in several solid tumors, such as gastric cancer, breast cancer, and colorectal cancer, as a biomarker for diagnostic and prognostic applications. However, there is still much work to do in the future on the other solid tumors such as lung cancer and pancreatic cancer, which have insufficient evidence in clinical diagnostic and prognostic applications of miR-27a. We found that there's little research of miR-27a in clinical study on pancreatic cancer or lung cancer while there's a possibility of linkage cause miR-27a has a profound influence in signaling pathways of these cancers. Further research directions could be toward these areas. What's more, though increasing the effectiveness of diagnosis and/or prognosis, the shortcoming of miR-27a still exists. Limited by the popularity of instrumentation and experimental technology, we failed to find the current clinical trials based on miR-27a as a biomarker compared to traditional biomarkers such as carbohydrate antigen. MicroRNA also has a significant heterogeneity of expression level, leading to more obstacle in clinical using, for example, hypoxia can infect the expression level of microRNA in different parts of the tumor, leading to a different examine result, which may bring difficulties in diagnosis (18, 125, 126). In reality, biopsy samples tend to probe one specific area and do not provide more details into the dynamics of miRNA expression in solid tumors, which brings further problems in the application. Nevertheless, using microRNAs as a therapeutic or diagnostic method has progressed from bench to bedside, with some successful phase I and/or phase II trials in cancer (NCT01829971, NCT02369198). Furthermore, more economical and effective methods of microRNA capture give us precise identification of the miRNA targetsome such as miR-CLIP seq. The mature database of microRNA brings us convenience in researching the potential pathways. We are looking forward to more clinical trials of miR-27a put into practice. We are also expecting further research on drug design based on miR-27a since there are emerging molecular closing related to miR-27a expression come out.

## Author Contributions

JZ was the first author of this review. ZC contributed equally to this review. All authors read and approved the final manuscript.

### Conflict of Interest Statement

The authors declare that the research was conducted in the absence of any commercial or financial relationships that could be construed as a potential conflict of interest.

## Abbreviations

RISC: RNA-induced silencing complex
EMT: epithelial-mesenchymal transition
AGO2: Argonaute 2
XPO5: exportin 5
PHLPP2: pleckstrin homology domain and leucine-rich repeat protein phosphatase 2
FOXO1: Forkhead box protein O1
SFRP: secreted frizzled-related protein
HCC: hepatocellular carcinoma
BTG2: B cell translocation gene 2
FBXW7: F-Box and WD Repeat Domain Containing 7 gene
TGF-β: tumor growth factor beta
TMEM170B: Transmembrane protein 170B
PPAR: peroxisome proliferator activated-receptors
DUSP16: dual specificity phosphatase 16
UPS: ubiquitin-proteasome system
BCSLCs: breast cancer stem-like cells
NSCLC: non-small cell lung cancer
CAFs: carcinoma-associated fibroblasts
FZD7: Frizzled Class Receptor 7
TNBC: triple-negative breast cancer.
